# Association between Serum Bilirubin and Estimated Glomerular Filtration Rate among Diabetic Patients

**DOI:** 10.1155/2015/480418

**Published:** 2015-01-26

**Authors:** Takeaki Katoh, Ryuichi Kawamoto, Katsuhiko Kohara, Tetsuro Miki

**Affiliations:** ^1^Department of Community Medicine, Ehime University Graduate School of Medicine, Ehime 791-0295, Japan; ^2^Department of Geriatric Medicine, Ehime University Graduate School of Medicine, Ehime 791-0295, Japan

## Abstract

The subjects comprised 230 men aged 77 ± 10 (range: 50–100) years and 279 women aged 81 ± 10 (50–101) years that visited the medical department. We examined the relationship between increased serum bilirubin and renal function evaluated by estimated glomerular filtration rate (eGFR) using CKD-EPI equations modified by a Japanese coefficient. Compared with the fourth quartile in serum bilirubin (1.01–1.97 mg/dL), the nonadjusted, age and gender-adjusted, and multivariate-adjusted odds ratios {95% confidence interval (CI)} of eGFR <60 mL/min/1.73 m^2^ for the first quartile in serum bilirubin (0.13–0.50 mg/dL) were 2.08 (1.25–3.44), 1.82 (1.07–3.09), and 1.53 (0.83–2.81), respectively. Moreover, compared with the fourth quartile, nonadjusted, age and gender-adjusted, and multivariate-adjusted odds ratios (95% CI) of eGFR <45 mL/min/1.73 m^2^ for the first quartile were 3.50 (1.95–6.23), 3.12 (1.72–5.65), and 3.53 (1.71–7.26), respectively. The data were further stratified by gender, age, medication (antihypertensive, antidyslipidemic, and antidiabetic agents), and prevalence of cardiovascular disease (CVD). The standardized coefficients for eGFR were significant in all the subgroups other than the prevalence of CVD, and there were significant interactions between the two groups regarding CVD. Our data demonstrated an independent positive association between serum bilirubin and eGFR among diabetic patients.

## 1. Introduction

Serum bilirubin may protect against inflammation, cardiovascular disease (CVD), and all-cause mortality in adults [1, 2]. Moreover, current evidences demonstrate that mildly elevated serum bilirubin may confer potent antioxidant properties, as indicated by its ability to scavenge peroxyl radicals and to inhibit oxidation of low-density lipoprotein (LDL) derived lipids [3, 4]. Lots of studies have shown a positive relationship between serum bilirubin and estimated glomerular filtration rate (eGFR) [5–9], showing that serum bilirubin has a potential renoprotective effect. We also demonstrated an independent positive association between serum bilirubin and eGFR in both genders among elderly persons [10]. Therefore, it is reasonable to speculate that serum bilirubin levels may be negatively correlated with diabetic nephropathy and renal function among diabetic patients.

Several cross-sectional studies have shown that low serum bilirubin levels were significantly associated with decreased eGFR, and negatively associated with diabetic nephropathy in a hospital-based sample of diabetic patients [6, 7]. In a cohort of Japanese type 2 diabetic patients, Mashitani et al. [9, 11] demonstrated that serum bilirubin levels were prospectively associated with diabetic nephropathy progression, independent of possible confounders. In contrast, Targher et al. [12] found that serum bilirubin was negatively associated with eGFR, considering serum bilirubin as a renal risk factor. Thus, a relationship between serum bilirubin and renal function remains controversial.

We evaluated the relationship of serum bilirubin with confounding risk factors such as renal function, as well as hypertension, hyperglycemia, and lipids, using cross-sectional data from the Nomura study [10].

## 2. Methods

### 2.1. Subjects

Patients for this investigation were recruited among consecutive diabetic patients aged ≥50 years that visited the Medical Department of Seiyo Municipal Nomura Hospital. Patients with serum bilirubin > 2.0 mg/dL and severe cardiorenal (e.g., Gilbert's syndrome) or nutritional disorders that would affect blood pressure, lipid, and glucose metabolism were excluded. Thus, 509 persons were enrolled in the study. All procedures were approved by the Ethics Committee of Seiyo Municipal Nomura Hospital, and written informed consent was obtained from each patient.

### 2.2. Evaluation of Confounding Factors

Information on demographic characteristics and confounding factors was collected using clinical files in all cases. Body mass index (BMI) was calculated by dividing weight (in kilograms) by the square of the height (in meters). We measured systolic blood pressure (SBP) and diastolic blood pressure (DBP) in the right upper arm of patients while in a sedentary position using a standard sphygmomanometer or an automatic oscillometric blood pressure recorder. Smoking status was quantified based on daily consumption and duration of smoking (pack·year) irrespective of the difference between current and past smoking status: never, light (<20 pack·year), moderate (20–39 pack·year), and heavy (≥40 pack·year). Total cholesterol (T-C), triglycerides (TG), high-density lipoprotein cholesterol (HDL-C), fasting plasma glucose (FPG), creatinine (enzymatic method), uric acid, and serum bilirubin were measured during a fasting condition within 24 hours after admission. Low-density lipoprotein cholesterol (LDL-C) level was calculated by the Friedewald formula [13], and those patients with TG levels ≥400 mg/dL were excluded. eGFR was calculated using CKD-EPI equations modified by a Japanese coefficient (eGFR_CKDEPI_): male, Cr ≤ 0.9 mg/dL, 141 × (Cr/0.9)^−0.411^ × 0.993^age^ × 0.813; Cr > 0.9 mg/dL, 141 × (Cr/0.9)^−1.209^ × 0.993^age^ × 0.813; female, Cr ≤ 0.7 mg/dL, 144 × (Cr/0.7)^−0.329^ × 0.993^age^ × 0.813; Cr > 0.7 mg/dL, 144 × (Cr/0.7)^−1.209^ × 0.993^age^ × 0.813 [14]. Histories of antihypertensive, antidyslipidemic, and antidiabetic medication use were also evaluated. Moreover, ischemic stroke, ischemic heart disease, and peripheral vascular disease were defined as CVD.

### 2.3. Statistical Analysis

All values are expressed as the mean ± standard deviation (SD), unless otherwise specified, and in the cases of parameters with nonnormal distribution (such as TG, FPG, and serum bilirubin), the data are shown as median (interquartile range) values. In all the analyses, parameters with nonnormal distributions were used after log-transformation. Statistical analysis was performed using IBM SPSS Statistics Version 21 (Statistical Package for Social Science Japan, Inc., Tokyo, Japan). Pearson's correlations were calculated in order to characterize the associations between various characteristics and eGFR. A multiple regression model was employed to evaluate the contribution of each confounding factor to eGFR. Subjects were divided into four groups based on the stage of eGFR (stage 1, eGFR ≥ 90; stages 2, 89.9 to 60; stage 3a, 59.9 to 45.0; stage 3b, 44.9 to 30.0; stage 4, <30 mL/min/1.73 m^2^) and quartile of serum bilirubin (Q-1, 0.13–0.50; Q-2, 0.51–0.70; Q-3, 0.71–1.00; Q-4, 1.01–1.97 mg/dL), and logistic regression analyses were used to test significant determinants of CKD serving as the dichotomous outcome variable. To examine the consistency of the observed association between serum bilirubin levels and eGFR, we performed subgroup analyses by age (<80, ≥80 years), medication (such as antihypertensive, antidyslipidemic, and antidiabetic agents) (absence, presence), serum uric acid (first-second tertiles, third tertile), and CVD (absence, presence), and interaction between serum bilirubin and the subgroups was analyzed by a general linear model. A value of *P* < 0.05 was considered significant.

## 3. Results

### 3.1. Subject Background Factors of the Subjects

Table 1 shows the value of background factor of the subjects. The subjects comprised 230 men aged 77 ± 10 (range, 50–100) years and 279 women aged 81 ± 10 (range, 50–101) years. Mean uric acid, Cr, and eGFR in the study sample were 5.5 ± 2.1 (SD) mg/dL, 1.1 ± 0.9 mg/dL, and 56.0 ± 20.2 mL/min/1.73 m^2^ with 1.0% stage 1, 51.5% stage 2, 21.2% stage 3a, 14.5% stage 3b, and 12.8% stage 4, respectively. Median serum bilirubin level was 0.7 (interquartile range, 0.5–1.0) mg/dL. Prevalence of antihypertensive, antidyslipidemic, antidiabetic medication, and CVD was 56.2%, 8.4%, 43.6%, and 40.1%, respectively.

### 3.2. Relationship of Risk Factors, including Serum Bilirubin and eGFR

**Figure 1:**
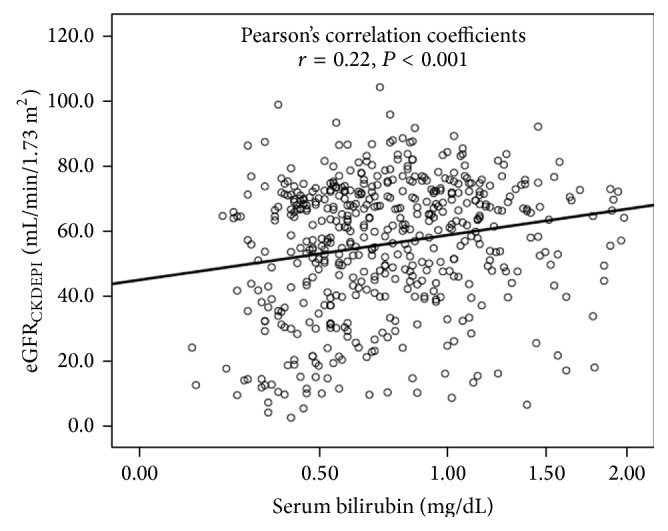
Relationship between serum bilirubin and estimated glomerular filtration rate (eGFR). CKD-EPI equations modified by a Japanese coefficient: male, Cr ≤ 0.9 mg/dL, 141 × (Cr/0.9)^−0.411^ ×  0.993^age^  ×  0.813; Cr > 0.9 mg/dL, 141 × (Cr/0.9)^−1.209^  × 0.993^age^  ×  0.813; female, Cr ≤ 0.7 mg/dL, 144 × (Cr/0.7)^−0.329^× 0.993^age^  ×  0.813; Cr > 0.7 mg/dL, 144 × (Cr/0.7)^−1.209^× 0.993^age^  × 0.813. Serum bilirubin was skewed and log-transformed for analysis.

Table 2 shows the relationship between participant characteristics and eGFR. Serum bilirubin (*r* = 0.22, *P* < 0.001) along with age, DBP, prevalence of antihypertensive medication, TG, HDL-C, LDL-C, and serum uric acid correlated significantly with eGFR (Figure 1). Stepwise multiple regression analysis using eGFR as an objective variable, adjusted for risk factors as explanatory variables, showed that serum bilirubin (*β* = 0.13, *P* < 0.001) was significantly and independently associated with eGFR, in addition to gender, age, prevalence of antihypertensive medication, HDL-C, and serum uric acid.

### 3.3. Relationship between Serum Bilirubin Categories and Risk for Reduced eGFR (Stages 3 + 4 or Stages 3b + 4)

Table 3 shows the odds ratio of renal dysfunction for each quartile increase in serum bilirubin. eGFR values decreased significantly and progressively with decreasing serum bilirubin. The prevalence of eGFR < 60 mL/min/1.73 m^2^ (stages 3 + 4) for each quartile in serum bilirubin was 40.8% Q-4, 43.2% Q-3, 51.1% Q-2, and 58.9% Q-1, respectively. Compared with Q-4 in serum bilirubin, nonadjusted, age and gender-adjusted, and multivariate-adjusted odds ratios {95% confidence interval (CI)} of stages 3 + 4 for Q-1 in serum bilirubin were 2.08 (1.25–3.44), 1.82 (1.07–3.09), and 1.53 (0.83–2.81), respectively. Moreover, the prevalence of eGFR < 45 mL/min/1.73 m^2^ (stages 3b + 4) for each quartile in serum bilirubin was 17.6% Q-4, 21.6% Q-3, 27.4% Q-2, and 42.7% Q-1, respectively. Compared with Q-4, nonadjusted, age and gender-adjusted, and multivariate-adjusted odds ratios (95% CI) of stages 3b + 4 for Q-1 were 3.50 (1.95–6.23), 3.12 (1.72–5.65), and 3.53 (1.71–7.26), respectively.

### 3.4. Relationship of Serum Bilirubin and eGFR within Selected Subgroups

Next, to control potential confounding factors, the data were further stratified by gender, age, medication (antihypertensive, antidyslipidemic, and antidiabetic agents), serum uric acid (first-second tertiles, third tertile), and prevalence of CVD (Table 4). The standardized coefficients for eGFR were significant in all subgroups other than the prevalence of CVD, and there were significant interactions only between the two groups regarding CVD.

## 4. Discussion

To examine the possible contribution of decreased serum bilirubin to renal dysfunction among diabetic persons, we studied the relationship between potential confounding risk factors including serum bilirubin and eGFR. This study showed a graded decrease in eGFR with decreasing serum bilirubin. Individuals with hypobilirubinemia (first quartile of serum bilirubin,** <**0.50 mg/dL) showed increased risk for stage 3b CKD (eGFR < 45 mL/min/1.73 m^2^). Moreover, the strength of serum bilirubin level as an independent determinant of eGFR was similar to those of known factors such as gender, age, prevalence of antihypertensive medication, HDL-C, and serum uric acid. To our knowledge, few epidemiologic studies have quantified the link between decreased serum bilirubin and renal dysfunction in diabetic patients. Thus, we think that serum bilirubin levels may be utilized as a provisional new confounding factor of diabetic nephropathy that can be measured easily and applied in medical practice.

Several studies have shown that decreased serum bilirubin is a risk factor for the development of CKD among type 2 diabetic individuals. Inoguchi et al. [15] showed a lower prevalence of vascular complications as well as reduced markers of oxidative stress and inflammation in patients with Gilbert's syndrome, which is a congenital hyperbilirubinemia, and diabetes. A community-based cross-sectional study in Korea [7] found that total serum bilirubin levels were negatively correlated with 24-hour proteinuria and positively associated with eGFR after adjusting for potential confounding factors in 612 diabetic patients. Fukui et al. [6] found that serum bilirubin level was independently and negatively associated with albuminuria in a hospital-based cross-sectional study in 633 Japanese type 2 diabetic patients. In addition, it was shown that serum bilirubin levels were higher in patients without diabetic nephropathy than in those with diabetic nephropathy. In a longitudinal cohort study of 12,823 Korean male workers without CKD or proteinuria at baseline, higher serum direct bilirubin levels were significantly associated with a lower risk of developing CKD (eGFR, <60 mL/min/1.73 m^2^), even after adjusting for potential confounding factors [5]. In the single-center longitudinal observational cohort study of type 2 diabetic patients, Toya et al. [11] found that higher serum bilirubin levels, within the normal range, were associated with a lower risk of progression from microalbuminuria to macroalbuminuria. In a hospital-based study of 2,678 US diabetic outpatients (mean age: 55 ± 18 years), Targher et al. [12] found that serum bilirubin levels were negatively associated with eGFR. However, in that study, no adjustment was made for potential confounding factors. Longitudinal data from 2,511 type 2 diabetic Japanese patients showed that multivariable-adjusted odds ratios for progression from microalbuminuria to macroalbuminuria for the second, third, and fourth quartiles of serum bilirubin levels were 0.89 (95% CI 0.49–1.58), 0.93 (0.47–1.83), and 0.33 (0.13–0.84), respectively. However, this trend disappeared after adjustment for hemoglobin level [9]. In our hospital-based sample of 509 individuals, we found that decreased serum bilirubin levels were significantly associated with decreased eGFR, and multivariate adjusted-odds ratio of hypobilirubinemia (0.13–0.50 mg/dL) for stage 3b (eGFR < 45 mL/min/1.73 m^2^) was 3.53 (1.71–7.26).

The mechanism by which serum bilirubin level is associated with a lower risk of CKD is not completely understood. Bilirubin has been described as the most powerful endogenous antioxidant substance in vitro [16] when acting alone and complexed with serum albumin to serve as a superoxide scavenger and peroxyl radical trapping antioxidant [17]. Hyperglycemia causes mitochondrial superoxide overproduction in vascular endothelial cells [18]. A recent study in a rodent model discovered a protective effect of bilirubin against diabetic nephropathy through inhibition of renal nicotinamide adenine dinucleotide phosphate- (NADPH-) dependent superoxide production and both hyperglycemia and angiotensin-II-induced production of reactive oxygen species [19]. Taken together, the present results suggest that bilirubin might have a protective role in the progression of diabetic nephropathy, particularly in diabetic patients with greater oxidative stress such as CVD. In our study, the standardized coefficient for eGFR was significant in the subgroup with prevalence of CVD, and there were significant interactions between the two groups regarding CVD.

We must be aware of the limitations to the present study. First, due to the cross-sectional study design, the present results are inherently limited in the ability to eliminate causal relationships between serum bilirubin and eGFR among diabetic patients. Second, our definition of eGFR is based on a single assessment of serum creatinine, which may introduce a misclassification bias. Third, estimating GFR listed as the CKD-EPI equation tends to be less accurate in subjects with normal renal function and CKD than GFR when inulin clearance is used, but is more accurate than serum creatinine or eGFR using the Modification of Diet in Renal Disease (MDRD) formula [14]. Fourth, in this study, CKD may have been misclassified with eGFR > 60 mL/min/1.73 m^2^ and proteinuria as mildly reduced renal function because renal dysfunction was defined as reduced eGFR irrespective of the presence or absence of proteinuria. Therefore, the demographics and referral source may limit generalizability.

## 5. Conclusion

The present study showed that decreased serum bilirubin levels are strongly associated with decreased eGFR among diabetic patients. The underlying mechanism behind this relationship is unclear, but seems to be independent of traditional confounding risk factors such as age, hypertension, and dyslipidemia. For community-dwelling diabetic patients, prospective population-based studies are needed to investigate the mechanisms underlying this association.

## Figures and Tables

**Table 1:** Characteristics of various risk factors of the subjects.

Characteristic (*N* = 509)	Value
Gender male (%)	45.2
Age (years)	79 ± 10
Body mass index^†^ (kg/m^2^)	21.5 ± 3.9
Smoking status^‡^ (%)	74.7/2.2/9.8/13.4
Systolic blood pressure (mmHg)	137 ± 27
Diastolic blood pressure (mmHg)	75 ± 15
Antihypertensive medication (%)	56.2
Triglycerides (mg/dL)	82 (61–114)
HDL cholesterol (mg/dL)	55 ± 17
LDL cholesterol (mg/dL)	104 ± 35
Antidyslipidemic medication (%)	8.4
Fasting blood glucose (mg/dL)	151 (132–183)
Antidiabetic medication (%)	43.6
Serum uric acid (mg/dL)	5.5 ± 2.1
Serum creatinine (mg/dL)	1.1 ± 0.9
eGFR (mL/min/1.73 m^2^)	56.0 ± 20.2
CKD stage (1 + 2/3a/3b/4), %	51.5/21.2/14.5/12.8
Serum bilirubin (mg/dL)	0.7 (0.5–1.0)
Cardiovascular disease (%)	40.1

Data are presented as means ± standard deviation. HDL: high-density lipoprotein; LDL: low-density lipoprotein; eGFR: estimated glomerular filtration rate. ^†^Body mass index was calculated using weight in kilograms divided by the square of the height in meters. ^‡^Smoking status: daily consumption (pack) × duration of smoking (year) {never, light (<20 pack·year), moderate (20–39 pack·year), and heavy (≥40 pack·year)}. Data for triglycerides, fasting plasma glucose, and serum bilirubin were skewed and are presented as median (interquartile range) values.

**Table 2:** Relationship between various risk factors including serum bilirubin and estimated glomerular filtration rate.

	Pearson's	Multiple linear regression analysis	
Characteristic (*N* = 509)	correlation	Forced method	Stepwise method
	*r* (*P* value)	*β* (*P* value)	*β* (*P* value)
Gender (male = 0, female = 1)	−0.08 (0.059)	−0.11 (0.006)	−0.07 (0.032)
Age	−0.35 (<0.001)	−0.29 (<0.001)	−0.27 (<0.001)
Body mass index	−0.02 (0.645)	−0.03 (0.397)	—
Smoking status	0.02 (0.738)	−0.06 (0.129)	—
Systolic blood pressure	0.05 (0.260)	—	—
Diastolic blood pressure	0.21 (<0.001)	0.05 (0.174)	—
antihypertensive medication	−0.18 (<0.001)	−0.08 (0.026)	−0.09 (0.004)
Triglycerides	−0.17 (<0.001)	−0.06 (0.152)	—
HDL cholesterol	0.14 (0.002)	0.06 (0.077)	0.09 (0.008)
LDL cholesterol	0.11 (0.017)	0.07 (0.057)	—
Antidyslipidemic medication	0.00 (0.974)	−0.02 (0.573)	—
Fasting blood glucose	0.03 (0.501)	0.01 (0.736)	—
Antidiabetic medication	0.00 (0.983)	−0.04 (0.233)	—
Serum uric acid	−0.59 (<0.001)	−0.53 (<0.001)	−0.56 (<0.001)
Serum bilirubin	0.22 (<0.001)	0.12 (<0.001)	0.13 (<0.001)
*R* ^2^	—	0.50 (<0.001)	0.49 (<0.001)

*r*: Pearson's correlation coefficient; *β*: standardized coefficient; *R* ^2^: multiple coefficient of determination. Data for triglycerides, fasting plasma glucose, and serum bilirubin were skewed and log-transformed for analysis.

**Table 3:** Relationship between serum bilirubin categories and risk for reduced eGFR.

Characteristic *N* = 509	Quartiles of serum bilirubin (mg/dL)				*P* value
	Q-4 1.01–1.97 *N* = 125	Q-3 0.71–1.00 *N* = 125	Q-2 0.51–0.70 *N* = 135	Q-1 0.13–0.50 *N* = 124	
eGFR (mL/min/1.73 m^2^)	59.5 ± 17.9	60.2 ± 18.8	56.0 ± 18.9	48.1 ± 22.9	<0.001
Prevalence of eGFR <60, *N* (%)	51 (40.8)	54 (43.2)	69 (51.1)	73 (58.9)	0.018
Nonadjusted OR (95% CI)	1.00	1.10 (0.67–1.82)	1.52 (0.93–2.48)	2.08 (1.25–3.44)	0.018
Age and gender adjusted OR (95% CI)	1.00	1.23 (0.72–2.09)	1.52 (0.91–2.54)	1.82 (1.07–3.09)	0.133
Multivariate adjusted OR (95% CI)	1.00	1.30 (0.70–2.41)	1.48 (0.81–2.72)	1.53 (0.83–2.81)	0.514
Prevalence of eGFR <45, *N* (%)	22 (17.6)	27 (21.6)	37 (27.4)	53 (42.7)	<0.001
Nonadjusted OR (95% CI)	1.00	1.29 (0.69–2.42)	1.77 (0.97–3.21)	3.50 (1.95–6.25)	<0.001
Age and gender adjusted OR (95% CI)	1.00	1.33 (0.70–2.54)	1.71 (0.93–3.14)	3.12 (1.72–5.65)	0.001
Multivariate adjusted OR (95% CI)^§^	1.00	1.68 (0.78–3.64)	1.74 (0.83–3.65)	3.53 (1.71–7.26)	0.004

CKD: chronic kidney disease: OR: odds ratio; CI: confidence interval. ^§^Adjusted for all confounding factors in the stepwise method in Table 2 by multiple logistic regression analysis. Data for triglycerides were skewed and log-transformed for analysis.

**Table 4:** Relationship between serum bilirubin and estimated glomerular filtration rate within selected subgroups.

Characteristics (*N* = 509)	*N*	*β* (*P* value)	*P*-interaction
Gender			
Men	230	0.13 (0.013)	0.923
Women	279	0.14 (0.001)	
Age			
<80 years	200	0.21 (0.001)	0.075
≥80 years	279	0.10 (0.028)	
Medication			
Absence	141	0.12 (0.047)	0.670
Presence	368	0.14 (<0.001)	
Serum uric acid			
First-second tertiles	319	0.18 (<0.001)	0.830
Third tertile	190	0.13 (<0.001)	
Cardiovascular disease			
Absence	305	0.05 (0.210)	0.020
Presence	204	0.21 (<0.001)	

*β*: standardized coefficient. Medication included antihypertensive, antidyslipidemic, and antidiabetic agents. ^§^Adjusted for all confounding factors in the stepwise method in Table 2 by multiple linear regression analysis.
